# Identification of actin mutants with neurodegenerative disease‐like phenotypes via mutagenesis of the actin‐ATP interface

**DOI:** 10.1002/alz70861_108589

**Published:** 2025-12-23

**Authors:** Keerthana Surabhi, Mary Phipps, Maelee Becton, Jahiem Hill, Robert M Hughes, ERZSEBET Maria SZATMARI

**Affiliations:** ^1^ East Carolina University, Greenville, NC USA

## Abstract

**Background:**

Cofilin‐actin rods formation is frequently associated with neurodegenerative diseases, including Alzheimer’s disease and Parkinson disease. However, the role of specific actin residues in promoting the formation of abnormal cytoskeletal structures such as cofilin‐actin rods, is largely unknown. Given that specific mutations and post‐translational modifications of actin could promote neurodegeneration, understanding the role of these residues in the development of abnormal cytoskeletal dynamics is highly relevant.

**Method:**

In this study, we focused on the actin‐ATP interface, which has been proposed as a key mediator of cofilin‐actin rod formation and the propensity of actin to respond to cellular stress. Using a light and stress‐gated reporter of cofilin‐actin cluster formation, we evaluated the impact of mutants associated with Actin‐ATP binding on the propensity of actin to form anomalous structures in the presence and absence of cellular stress.

**Results:**

We identified actin mutants that promote anomalous actin inclusions and characterized the manifestation of these phenotypes in cortical neuron cultures. Mutations to the ATP phosphate tail‐binding region of actin (K18A, D154A, G158L, K213A) were found to be particularly disruptive to actin phenotypes, promoting the formation of disease‐associated actin‐rich structures (such as cofilin‐actin rods and Hirano bodies) and loss of dendritic spines without promoting neuronal death.

**Conclusions:**

Mutations to the ATP phosphate tail‐binding region of actin strengthen the association of residue‐specific changes in actin with large‐scale phenotypic and functional changes in the cytoskeleton, further implicating them in neurodegenerative disease progression.

